# Supplementary material for: Disease mapping: Geographic differences in population rates of interventional treatment for prostate cancer in Australia
Source: PLoS One. 2023 Nov 13;18(11):e0293954. doi: 10.1371/journal.pone.0293954 (PMC10642787; doi:10.1371/journal.pone.0293954)
Supplement: S1 Appendix — This file includes additional results mentioned in the text of the manuscript as well as the complete model outputs for the “Area-level associations” section and a detailed description of the methods and sensitivity analysis. (PDF) [file pone.0293954.s001.pdf]

Supplementary material

Results

Area-level associations

Table S1; Separation rate ratios (SRR), standard errors (SE), 95% confidence intervals, z-statistic and p-value from the GLMM fitted for radical prostatectomy. Results of the likelihood ratio tests reflecting the evidence for an association, the mixed effects model or the negative binomial (compared with the Poisson) in the multivariate model are provided in bold font. Final model AIC was 43,457.

	SRR	SE	95% CI	z	p
State or territory					**< 0.001**
NSW	1.3	0.039	1.23 - 1.38	8.69	< 0.001
Victoria	1.11	0.033	1.04 - 1.17	3.45	0.001
Queensland	1.25	0.035	1.18 - 1.32	7.86	< 0.001
WA	1.17	0.037	1.10 - 1.24	4.87	< 0.001
SA	1				
Tasmania	1.06	0.046	0.98 - 1.16	1.40	0.161
ACT	1.44	0.055	1.34 - 1.55	9.57	< 0.001
NT	0.52	0.044	0.44 – 0.61	-7.67	< 0.001
Index of relative socioeconomic advantage and disadvantage					**< 0.001**
Most disadvantaged	0.67	0.017	0.64 - 0.71	-15.6	< 0.001
Q2	0.74	0.017	0.71 - 0.78	-12.91	< 0.001
Q3	0.77	0.016	0.74 - 0.81	-12.25	< 0.001
Q4	0.85	0.017	0.81 - 0.88	-8.1	< 0.001
Most advantaged	1				
Remoteness					**< 0.001**
Major city	1				
Inner regional	1.08	0.021	1.04 - 1.12	3.81	< 0.001
Outer regional	1.04	0.029	0.99 - 1.10	1.42	0.156
Remote and very remote	0.8	0.046	0.72 - 0.90	-3.88	< 0.001
Age group					**< 0.001**
40-49	0.06	0.002	0.05 - 0.06	-159.06	< 0.001
50-59	0.39	0.004	0.39 - 0.40	-102.57	< 0.001
60-69	1				
70-79	0.53	0.006	0.51 - 0.54	-52.08	< 0.001
80+	0.05	0.002	0.05 - 0.06	-72.44	< 0.001
**Variance in SA2 RE**	0.06	0.004	0.06 - 0.07		**< 0.001**
**Conditional overdispersion**	0.02	1.12	0.02 – 0.03		**< 0.001**

Low dose rate brachytherapy

Table S2; Separation rate ratios (SRR), standard errors (SE), 95% confidence intervals, z-statistic and p-value from the GLMM fitted for low dose rate brachytherapy. Results of the likelihood ratio tests reflecting the evidence for an association, the mixed effects model or the negative binomial (compared with the Poisson) in the multivariate model are provided in bold font. Final model AIC was 20,168.

	SRR	SE	95% CI	z	p
**State or territory**					**< 0.001**
NSW	0.36	0.021	0.32 - 0.41	-17.63	< 0.001
Victoria	0.48	0.029	0.42 - 0.54	-12.32	< 0.001
Queensland	0.4	0.024	0.35 - 0.45	-15.03	< 0.001
WA	0.42	0.027	0.37 - 0.47	-13.51	< 0.001
SA	1				
Tasmania	0.51	0.062	0.40 - 0.65	-5.52	< 0.001
ACT	0.16	0.023	0.12 - 0.21	-12.56	< 0.001
NT	0.28	0.044	0.21 - 0.38	-8.17	< 0.001
**Index of relative socioeconomic advantage and disadvantage**					**< 0.001**
Most disadvantaged	0.53	0.035	0.47 - 0.60	-9.65	< 0.001
Q2	0.63	0.038	0.56 - 0.70	-7.8	< 0.001
Q3	0.70	0.040	0.63 - 0.79	-6.19	< 0.001
Q4	0.77	0.044	0.69 - 0.86	-4.57	< 0.001
Most advantaged	1				
**Remoteness**					**0.002**
Major city	1				
Inner regional	1.10	0.05	1.00 - 1.20	1.98	0.048
Outer regional	1.26	0.09	1.09 - 1.45	3.18	0.001
Remote and very remote	1.12	0.15	0.86 - 1.46	0.82	0.412
**Age group**					**< 0.001**
40-49	0.02	0.002	0.01 - 0.02	-39.11	< 0.001
50-59	0.27	0.008	0.26 - 0.29	-42.68	< 0.001
60-69	1				
70-79	1.03	0.028	0.97 - 1.08	1.01	0.313
80+	0.14	0.011	0.12 - 0.17	-25.67	< 0.001
**Variance in SA2 RE**	0.32	0.023	0.27 - 0.37		**< 0.001**
**Conditional overdispersion**	0.11	1.16	0.08 - 0.14		**< 0.001**

High dose rate brachytherapy

Table S3; Separation rate ratios (SRR), standard errors (SE), 95% confidence intervals, z-statistic and p-value from the GLMM fitted for high dose rate brachytherapy. Results of the likelihood ratio tests reflecting the evidence for an association and the mixed effects model in the multivariate model are provided in bold font. Final model AIC was 7143.

	SRR	SE	95% CI	z	p
State or territory					**< 0.001**
NSW	4.32	0.673	3.19 - 5.86	9.41	< 0.001
Victoria	1.14	0.195	0.81 - 1.59	0.75	0.454
Queensland	4.21	0.664	3.09 - 5.73	9.10	< 0.001
WA	0.36	0.088	0.22 - 0.58	-4.18	< 0.001
SA	1				
Tasmania	5.57	1.353	3.46 - 8.97	7.07	< 0.001
ACT	1.69	0.508	0.94 - 3.05	1.75	0.08
NT	1.55	0.880	0.51 - 4.72	0.78	0.436
Index of relative socioeconomic advantage and disadvantage					**< 0.001**
Most disadvantaged	0.48	0.050	0.40 - 0.59	-7.05	< 0.001
Q2	0.54	0.055	0.44 - 0.66	-6.03	< 0.001
Q3	0.55	0.053	0.46 - 0.67	-6.15	< 0.001
Q4	0.63	0.059	0.52 - 0.76	-4.92	< 0.001
Most advantaged	1				
Remoteness					**0.008**
Major city	1				
Inner regional	0.92	0.079	0.77 - 1.09	-1.01	0.313
Outer regional	0.66	0.080	0.52 - 0.84	-3.45	0.001
Remote and very remote	0.93	0.211	0.59 - 1.45	-0.32	0.746
Age group					**< 0.001**
40-49	0.02	0.004	0.01 - 0.03	-18.2	< 0.001
50-59	0.24	0.018	0.21 - 0.28	-18.92	< 0.001
60-69	1				
70-79	1.38	0.078	1.23 - 1.54	5.61	< 0.001
80+	0.22	0.033	0.16 - 0.29	-9.99	< 0.001
Variance in SA2 RE	0.36	0.048	0.28 - 0.47		**< 0.001**

Statistical groups

Table S4; Number and percentage of areas in each group by state, area-level socioeconomic quintile and remoteness category.

	High prostatectomy group	High EHR group	High LDR Brachytherapy group
**Total**	875 (41%)	739 (34%)	534 (25%)
State or territory			
New South Wales	378 (71%)	143 (27%)	13 (2%)
Victoria	76 (18%)	248 (58%)	104 (24%)
Queensland	304 (59%)	175 (34%)	38 (7%)
Western Australia	0 (0%)	50 (21%)	187 (79%)
South Australia	0 (0%)	12 (7%)	153 (93%)
Tasmania	15 (16%)	41 (43%)	39 (41%)
Australian Capital Territory	102 (97%)	3 (3%)	0 (0%)
Northern Territory	0 (0%)	67 (100%)	0 (0%)
Area-level socioeconomic index			
Most disadvantaged	119 (28%)	220 (53%)	80 (19%)
Q2	137 (33%)	159 (38%)	125 (30%)
Q3	151 (36%)	168 (40%)	100 (24%)
Q4	182 (43%)	123 (29%)	116 (28%)
Most advantaged	267 (63%)	54 (13%)	100 (24%)
Remoteness category			
Major city	589 (47%)	354 (29%)	299 (24%)
Inner regional	211 (43%)	163 (33%)	115 (24%)
Outer regional	67 (21%)	152 (47%)	102 (32%)
Remote and very remote	8 (8%)	70 (73%)	18 (19%)

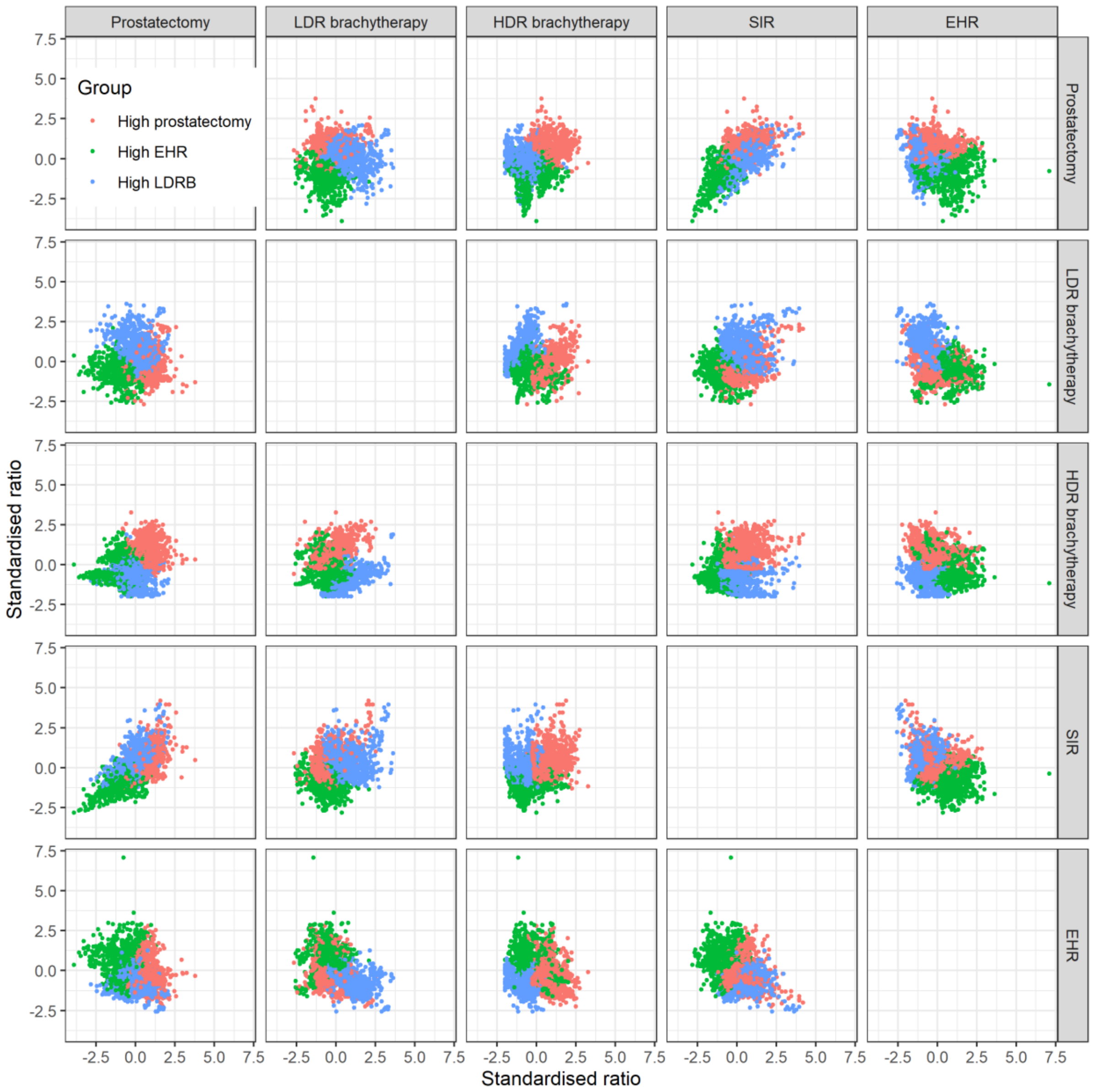

Figure S1; Pairwise scatterplots of area-level SSRs for the treatments for prostate cancer and standardised incidence ratios (SIRs) for prostate cancer diagnoses and excess hazard ratios (EHRs), with each dot in each subplot reflecting a single area and the symbol colour reflecting the area's statistical group. Note that the standardised ratios shown here have been normalised and log transformed prior to normalisation.

Methods

Medical coding

Table S5; ICD-10-AM/ACHI diagnosis related group codes for treatments for prostate cancer.

**Treatment**	**ICD-10-AM/ACHI codes**
High dose rate brachytherapy	15327-01, 15327-03, 15327-06, 15327-07, 15536-00, 15536-01, 15536-02
Low dose rate brachytherapy	15327-00, 15327-02, 15327-04, 15327-05, 15338-00
Radical prostatectomy	37209-00, 37210-00, 37211-00, 37209-01, 37210-01, 37211-01

Areal reallocation

This section describes in greater detail the reallocation from SLA to 2011 ASGS SA2. Let's describe the SLA as the "original area" (A_{Oi}) and the 2011 ASGS SA2s as the "final area" (A_{Fi}).

If area A_{Oi} overlaps $\boldsymbol{A_{Fi}} = \{A_{Fi1}, A_{Fi2}, \dots, A_{FiM}\}$, a set of M SA2s from the 2011 ASGS and the proportion of all individuals living in A_{Oi} who live in each area of $\boldsymbol{A_{Fi}}$ is

$$\boldsymbol{P_i} = \{P_{i1}, P_{i2}, \dots, P_{iM}\}$$

where $\sum_{m=1}^{M} P_{im} = 1$

Then we define the cumulative proportions as:

$$P'_{\mathrm{im}} = \sum_{v=1}^{m} P_{iv} \quad for\ all\ m\ in\ 1, \dots, M$$

Additionally, $P'_{i0} = 0$.

For each individual in the data set, a random number, $rand$, is generated using a uniform distribution between 0 and 1. An individual who was originally geocoded to A_{Oi} is then reallocated to A_{Fim}, such that $P'_{\mathrm{im}} \geq rand$ and $P'_{i(\mathrm{m}-1)} < rand$

Spatial modelling

For each of the procedures, the expected number of separations (E_i) was calculated for each SA2 (i, 2011 ASGS) for the ten-year period between 2007 and 2016, using the Australian total count (y_k) and population (pop_k) for each age group (k) and the population in the SA2 at each age group (pop_{ik}) as follows.

$$E_i = \sum_k \frac{y_k}{pop_k} pop_{ik}$$

Equation 1

The observed total number of separations in each SA2 (y_i) was modelled as a Poisson process with an intensity proportional to E_i.

$$y_i \sim Poisson(E_i \theta_i)$$

Equation 2

The log of the standardised separation rate ratio (θ_i) was a linear combination of an intercept (β_0) and a spatial random effect (S_i).

$$\log(\theta_i) = \beta_0 + S_i$$

Equation 3

A Leroux prior was applied to S_i, so that the spatial effect for area i depended on the spatial effects for all other areas ($S_{\setminus i}$), according to a neighbourhood matrix. The neighbourhood matrix was square with dimension equal to the number of SA2s being modelled. The $(i,j)^{\text{th}}$ element of the matrix (w_{ij}) was 1 if i^{th} and j^{th} SA2s were adjacent and 0 otherwise. The parameter ρ reflected the degree of spatial autocorrelation and σ_S^2 was the variance in the spatial random effects.

$$S_i | S_{\setminus i} \sim \mathcal{N}\left(\frac{\rho \sum_j w_{ij} S_j}{\rho \sum_j w_{ij} + 1 - \rho}, \frac{\sigma_S^2}{\rho \sum_j w_{ij} + 1 - \rho}\right)$$

Equation 4

Weakly informative priors were applied, as follows, where $\mathcal{IG}$ is the inverse gamma distribution and τ_S^2 was the precision in the spatial random effect.

$$\beta_0 \sim \mathcal{N}(0, 100\,000)$$

$$1/_{\sigma_S^2} = \tau_S^2 \sim \mathcal{IG}(1, 0.01)$$

$$\rho \sim Uniform(0, 1)$$

Equation 5

Convergence

Within chain convergence of the Markov chain Monte Carlo was confirmed using the Geweke diagnostic, with less than 10% of areas' having a significant Geweke diagnostic at the 5% level. Trace plots and posterior densities of the global parameters (β_0, ρ and σ_S) and a small number of area-specific parameters (θ_i and S_i) were visually inspected.

Sensitivity analysis

A sensitivity analysis was conducted to test whether the model was sensitive to choices of the parameter values for the priors on β_0 and τ_S^2 in Equation 5. The values trialled are shown in Table S6 and the results are shown in Figure S2, Figure S3 and Figure S4. Sensitivity analyses showed that the results were not sensitive to the choice of priors or hyperpriors. Similarly, the results were not sensitive to initial values or seed.

Table S6; Values for the hyperpriors trialled in the sensitivity analysis.

	Mean	Variance
β_0	-1; 0; 1; 2	50; 1000; 10 000; 100 000
	Scale	Shape
τ_S^2	0.00001; 0.001; 0.01; 0.1	1; 2; 3; 4

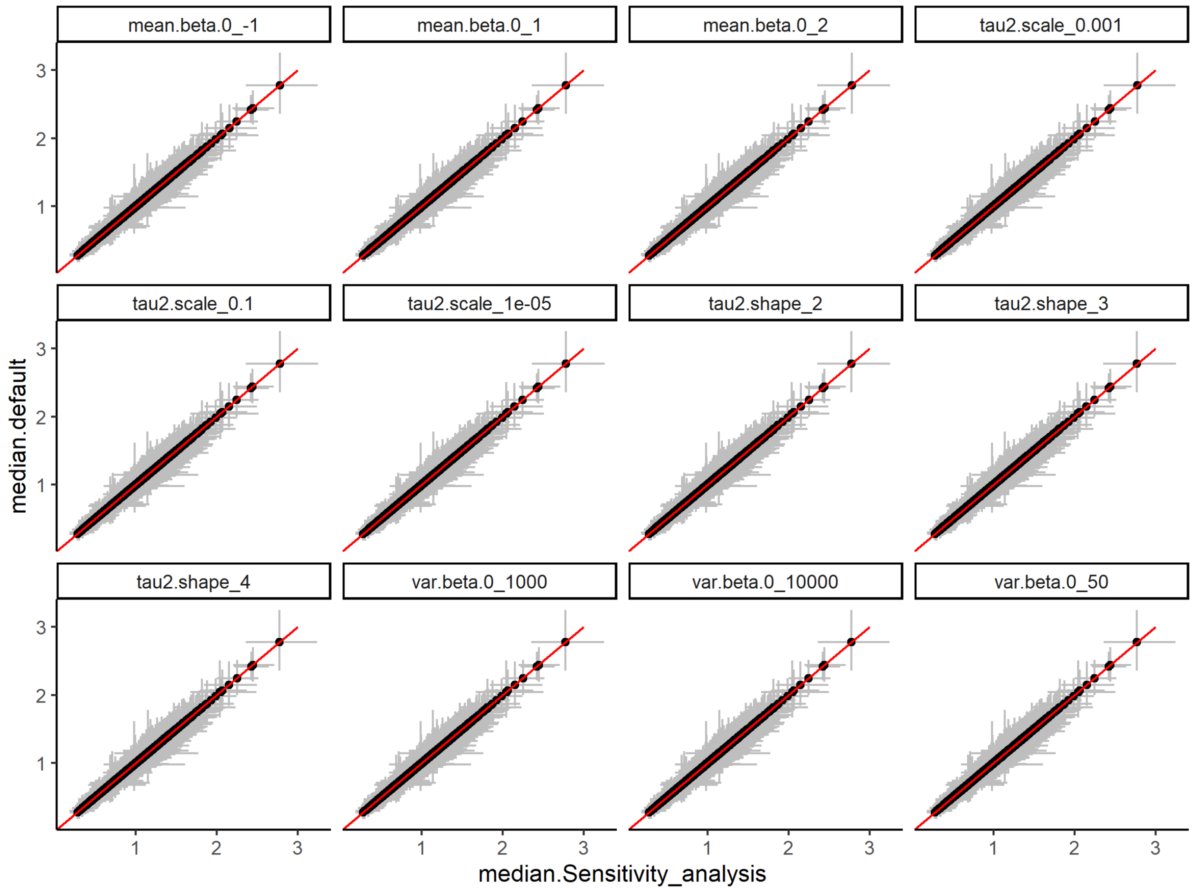

Figure S2; Results of the sensitivity analysis for radical prostatectomy. The y-axis shows the median SSR (and 95% credible interval) for each area using the default value for the hyperprior and the x-axis shows the median SSR (and 95% credible interval) for each area using the hyperprior value specified in the label for the subplot. The line of equality is shown in red to guide the eye.

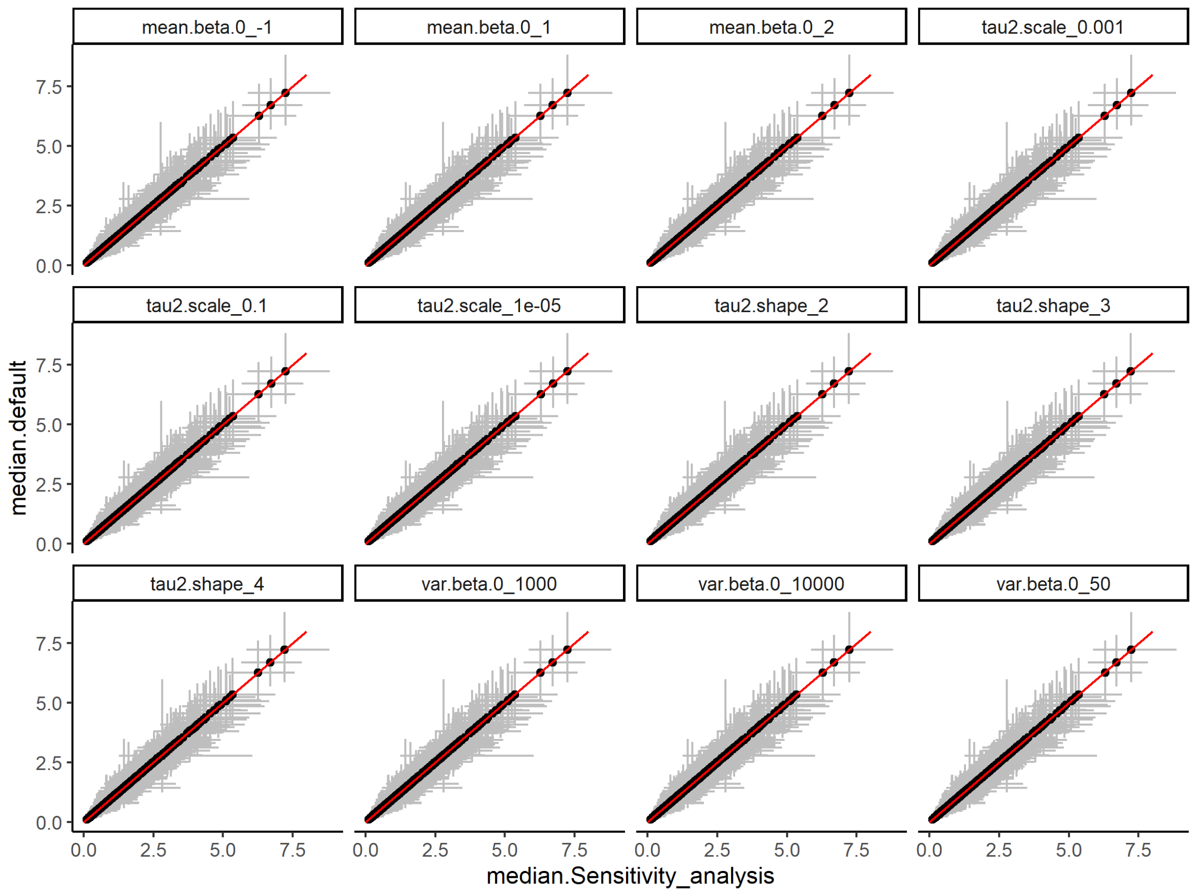

Figure S3; Results of the sensitivity analysis for low dose rate brachytherapy. The y-axis shows the median SSR (and 95% credible interval) for each area using the default value for the hyperprior and the x-axis shows the median SSR (and 95% credible interval) for each area using the hyperprior value specified in the label for the subplot. The line of equality is shown in red to guide the eye.

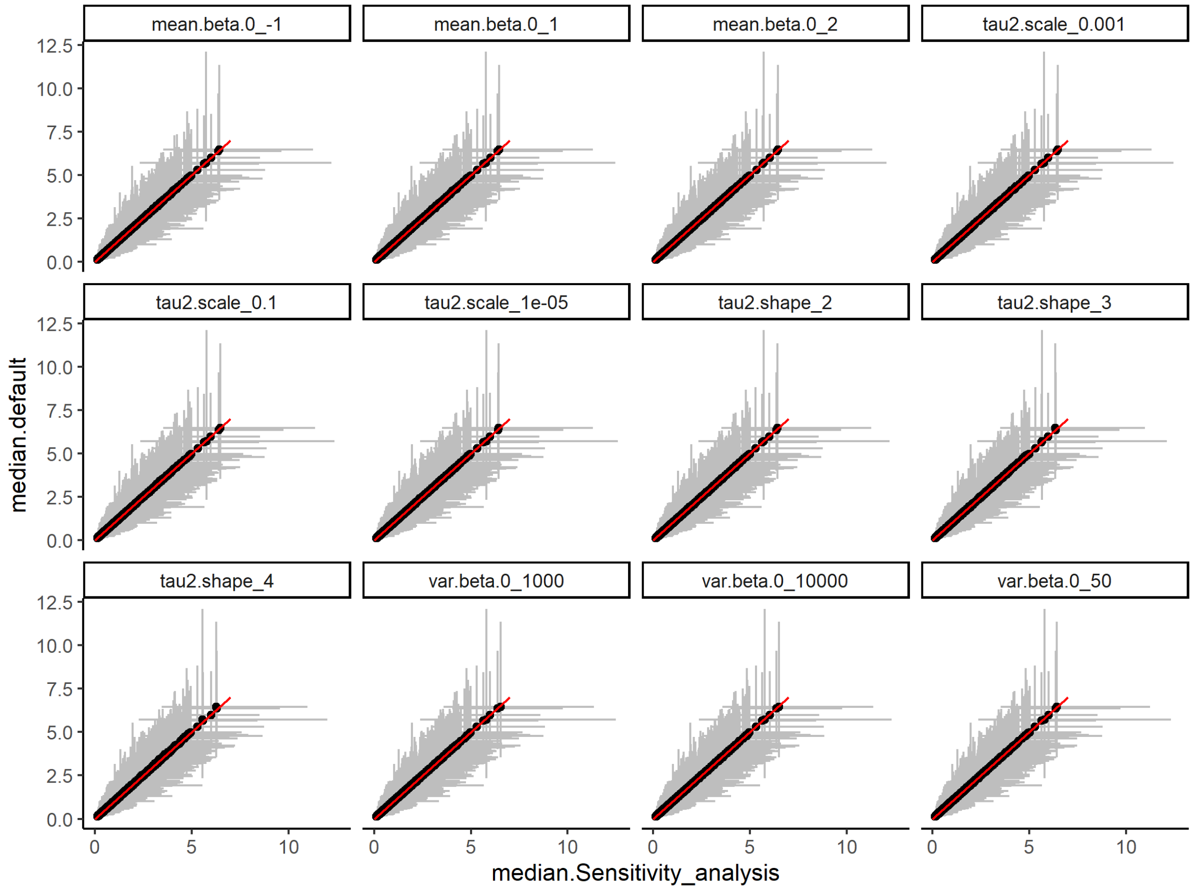

Figure S4; Results of the sensitivity analysis for high dose rate brachytherapy. The y-axis shows the median SSR (and 95% credible interval) for each area using the default value for the hyperprior and the x-axis shows the median SSR (and 95% credible interval) for each area using the hyperprior value specified in the label for the subplot. The line of equality is shown in red to guide the eye.
